# Design and Performance Evaluation of a Low-Cost High-SNR EOG Sensing System for Arabic Locked-In Syndrome Communication

**DOI:** 10.3390/s26082425

**Published:** 2026-04-15

**Authors:** Saleh I. Alzahrani, Najat Alomari, Sarah Alkilani, Lama Alghamdi, Bushra Melhem

**Affiliations:** Biomedical Engineering Department, College of Engineering, Imam Abdulrahman Bin Faisal University, Dammam 31451, Saudi Arabia; najat.alomary@gmail.com (N.A.); sarakilani_2000@hotmail.com (S.A.); lamaalghamdi24@gmail.com (L.A.); bmmulhem@iau.edu.sa (B.M.)

**Keywords:** electrooculogram (EOG), biosignal acquisition, biomedical sensor design, signal-to-noise ratio, assistive communication systems, locked-in syndrome, brain–computer interface, adaptive classification, low-cost sensing, neurorehabilitation technology

## Abstract

Locked-in Syndrome (LIS) is a neurological condition in which individuals remain conscious but experience complete paralysis of voluntary muscles, except for eye movements—highlighting the need for reliable assistive communication technologies. This study presents the design and evaluation of an Arabic electrooculogram (EOG)-based communication system with adaptive classification capabilities for LIS applications. A custom-designed EOG acquisition circuit incorporating filtering and amplification stages was implemented and compared with the OpenBCI Cyton board. The system employed a hybrid classification approach combining amplitude, temporal, and statistical features to distinguish between blinks and voluntary vertical eye movements. Testing with ten healthy subjects yielded a mean classification accuracy of 83.96% ± 4.59% and an information transfer rate of 10.43 letters per minute, corresponding to a 30.38% improvement over conventional approaches. The custom-designed circuit achieved a signal-to-noise ratio of 25.21 dB, outperforming the OpenBCI Cyton board by 8% while reducing system cost by 62%. The integration with a Morse code-based interface enabled Arabic letter composition, while the system incorporated auto-completion and text-to-speech functionalities to further enhance communication efficiency. This cost-effective solution addresses a critical gap in assistive technologies for Arabic-speaking individuals with LIS and shows strong potential for enhancing their communication abilities and overall quality of life.

## 1. Introduction

Locked-in Syndrome (LIS) is a severe neurological condition in which individuals remain conscious and cognitively intact but experience complete paralysis of voluntary muscles, except for eye movements and blinks [1,2]. This condition severely limits communication, significantly affecting quality of life and social interaction. One of the earliest fictional depictions of LIS appeared in the 19th century in Alexandre Dumas’s novel *The Count of Monte Cristo*, in which the character Monsignor Noirtier de Villefort relied exclusively on eye blinks for communication following cerebrovascular disease [3].

Clinically, the most common cause of LIS is cerebrovascular disease affecting the vertebrobasilar region. Other etiologies include tumors, viral infections, and traumatic injuries involving the brainstem or cerebellum [4]. Despite these severe physical impairments, individuals with LIS retain intact intellectual function and preserved linguistic capacity [5]. However, they are unable to communicate or express themselves through conventional motor or speech pathways, relying almost exclusively on eye movements and blinks. Additional symptoms may include episodes of involuntary emotional expression, such as pathological laughing or crying, reflecting dysregulated affective control. The mortality rate associated with LIS is particularly high during the first few months following onset [6]. Although complete recovery is rare, partial functional improvement may occur in some cases. The condition of individuals with LIS typically stabilizes within the first year, and life expectancy may extend over several years [7].

In recent decades, researchers have developed various computer-based communication techniques to enable alternative and augmentative communication for patients with LIS. Among these, eye-tracking technologies—such as scleral contact lenses and search coils, photo-oculography (POG), video-oculography (VOG), and electrooculography (EOG)—have shown promise in facilitating user interaction with external devices [8,9]. Although scleral search coils offer high accuracy, they are intrusive and often uncomfortable, while video-based eye trackers require sufficient lighting and significant computational power. In contrast, EOG has gained attention due to its low cost, compactness, and the ability to record eye movements independently of ambient lighting conditions, even when one’s eyes are closed. As EOG relies on detecting corneo-retinal electrical potential generated by eye movements relative to the head, it is especially suited for applications in assistive communication where minimal voluntary control remains. For LIS patients, who retain control of eye movements and blinks, EOG offers a non-invasive and efficient method for translating these actions into communicative commands. Despite the growing availability of assistive technologies, most are designed and optimized for European languages, leaving a significant gap in systems that support Arabic, the native language of more than 400 million speakers worldwide [10].

Current EOG-based communication systems are generally categorized into three main approaches: (1) virtual keyboard navigation, (2) predefined sentence selection, and (3) direct character formation. Each method offers distinct advantages and limitations. Virtual keyboard systems represent the most common approach in EOG-based communication [11]. Usakli and Gurkan developed a virtual keyboard system that achieved 95% accuracy and a processing speed of 12.24 letters per minute [12]. Their implementation utilized neural network techniques for eye movement classification but required substantial computational resources and relatively complex hardware configurations. Similarly, López et al. proposed a modular system with six functional units, including writing and email capabilities, which achieved a 40% improvement in processing speed compared to traditional systems [13]. However, this architectural complexity posed challenges for long-term usability.

An alternative approach involves predefined sentence selection. Banik et al. designed a system that used horizontal eye movements to select from ten predefined sentences, achieving high accuracy (92–99.6%) [14]. However, this method required numerous eye movements per selection, which reduced communication efficiency. To address this, Sugi et al. simplified the interface to only five selectable options, triggered through horizontal movements and blinks [15]. While more efficient, this severely limited communication flexibility.

More recently, research has explored direct character formation. Fang and Shinozaki introduced a system that enabled users to “write” Japanese Katakana characters using eye movements [16]. Although innovative, this method requires extensive user training and is less suitable for individuals with limited eye control. Their findings demonstrated the potential of language-specific solutions but also underscored the challenges of complex eye movement patterns. A comparative summary of representative EOG-based communication systems is provided in Table 1.

Across all approaches, accurate eye movement classification remains a critical challenge. Various techniques have been employed, including threshold-based methods, mathematical morphology, deep learning models, and hybrid models [17,18,19,20]. These techniques exhibit a wide range of accuracy levels (80–99%) and involve significant trade-offs between classification performance, computational cost, and system robustness. More recently, Hernández Pérez et al. applied wavelet-based feature extraction combined with KNN, SVM, and DT classifiers for EOG classification, reporting the best accuracy of 76.9% with SVM, underscoring the ongoing challenge of balancing accuracy and computational efficiency [21]. Gharkan et al. further demonstrated that combining deep feature extraction via a pre-trained ResNet50 model with ensemble learning on spectral and statistical EOG features achieves high classification accuracy for gaze direction; however, such deep learning approaches typically require substantially larger datasets and computational resources than threshold-based adaptive methods [22]. A comprehensive review by Belkhiria et al. surveying 112 EOG-based BCI publications from 2000 to 2020 found that despite significant technical advances, EOG systems remain predominantly designed for Latin-script applications, with limited support for other languages including Arabic [23].

Despite these advances, a critical analysis of existing systems reveals several significant limitations. First, most systems are tailored for the Latin alphabet or East Asian scripts, which provide minimal support for the unique visual and structural features of Arabic script [24]. Second, many systems demand complex movement patterns, contributing to user fatigue during prolonged use. Third, high-performance systems often rely on expensive hardware components, limiting their accessibility and scalability. Finally, few systems account for inter-individual variability in eye movement patterns or incorporate adaptive mechanisms to accommodate longitudinal changes in patient capability [25]. To address these limitations, we developed an optimized EOG-based communication system specifically tailored for Arabic-speaking individuals with LIS. The proposed system integrates cost-effective hardware with an adaptive classification algorithm to facilitate efficient, scalable, and accessible Arabic-language communication via eye movements.

Although the proposed system is built upon well-established analog signal conditioning stages, the main contribution of this work lies in a system-level design and optimization approach. Rather than introducing new circuit topologies, the study focuses on carefully integrating and tuning standard analog components to achieve high signal quality (SNR) using low-cost hardware. This cost–performance trade-off is a central objective, particularly for practical and accessible assistive technologies.

In addition, the system is specifically designed and evaluated for Arabic EOG-based communication, addressing a relatively underexplored area in BCI research. The proposed framework demonstrates that effective and reliable communication can be achieved through optimized engineering design without relying on complex or expensive hardware architectures. The cost-effectiveness of custom hardware has been validated in recent work; Kancaoğlu and Kuntalp demonstrated a low-cost mobile biosignal acquisition system using common integrated circuits that achieved biopotential signal quality comparable to commercial devices at significantly reduced cost, reinforcing the general feasibility of application-specific hardware design for BCI applications [26]. Notably, Tonin et al. successfully demonstrated an auditory EOG-based communication system directly in ALS patients transitioning from the locked-in to complete locked-in state, establishing that EOG-based systems can remain effective even as patients’ voluntary eye-movement amplitude decreases substantially—a finding directly relevant to the long-term applicability of the present system [27].

This paper is structured as follows: Section 2 outlines the methodology, including the participants, experimental protocol, signal acquisition, and classification approach. Section 3 presents the system performance results. Section 4 provides a detailed discussion of the findings. Finally, Section 5 concludes the paper and outlines directions for future research.

## 2. Materials and Methods

### 2.1. Participants

Ten healthy subjects (6 males and 4 females; mean age: 27.9 ± 4.7 years) were recruited for this study. All subjects had normal or corrected-to-normal vision and reported no history of neurological or ocular disorders that could affect eye movements. Each subject participated in an individual 30 min experimental session, which included a calibration phase, guided eye-movement tasks, and free-form communication trials. Written informed consent was obtained from all participants prior to the experimental sessions. The study protocol was approved by the Institutional Review Board (IRB) of Imam Abdulrahman Bin Faisal University (IAU).

### 2.2. Experimental Procedure

The experiment was conducted in a controlled laboratory environment under consistent lighting conditions and minimal external distractions. Each participant completed a single 30 min session that was divided into three phases: calibration, guided eye-movement tasks, and free-form communication trials. Participants were seated comfortably on adjustable chairs positioned approximately 50 cm from the mobile device display. The chair height and backrest angle were adjusted to ensure a natural, relaxed posture while keeping the eyes aligned with the center of the screen. Participants were instructed to rest their heads on a headrest to minimize head motion during signal acquisition. Before electrode placement, participants received standardized instructions about the three types of eye movements: regular blinks (natural eye closure lasting 100–300 ms), long blinks (deliberate, extended eye closure lasting more than 400 ms), and upward eye movements (directing the gaze upward toward the eyebrows). A demonstration video was presented to ensure consistent understanding across participants of each movement type.

**Figure 1:**
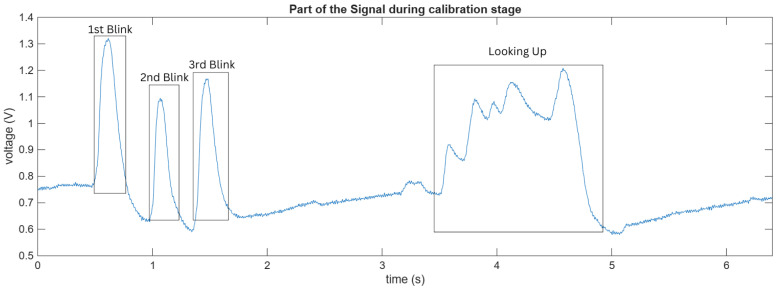
Recorded EOG signal during the calibration stage, illustrating multiple blink events and an upward eye movement. The figure highlights differences in signal amplitude and duration used for feature extraction and threshold definition.

Each session began with a personalized calibration phase designed to account for inter-individual variability in EOG amplitude and duration. Visual cues were displayed on the screen in the form of colored circles with corresponding text instructions. For regular blinks, a blue circle appeared with the text “BLINK NOW”; for long blinks, a green circle displayed “LONG BLINK”; and for upward movements, a red upward arrow was shown accompanied by “LOOK UP”. Each cue was displayed for 3 s, followed by a 2 s rest period during which a central fixation cross was shown. Participants performed four repetitions of each movement type (for a total of 12 trials) in a randomized order to minimize anticipation effects. The system recorded the amplitude, duration, and signal standard deviation from these calibration trials to establish participant-specific thresholds. For a better understanding of the calibration procedure, Figure 1 shows a representative segment of the recorded EOG signal. The figure illustrates typical signal patterns corresponding to different eye movements, including regular blinks and upward eye movement. These variations highlight the differences in amplitude and duration that are used to extract features and define classification thresholds during calibration.

**Figure 2:**
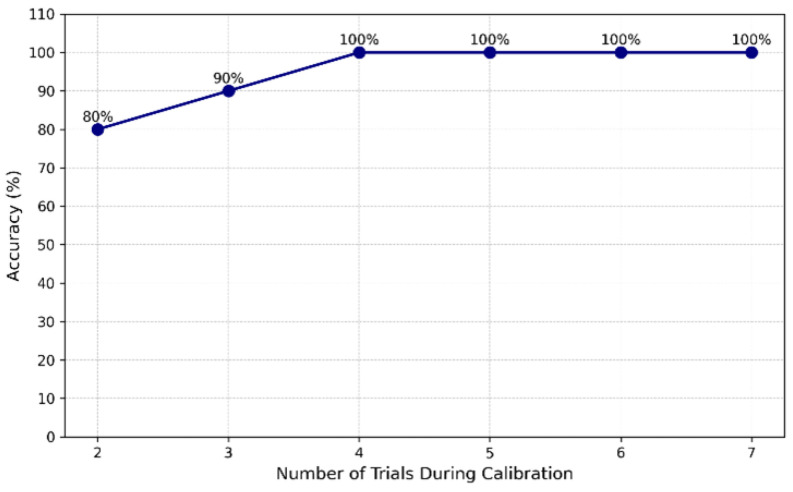
Classification accuracy as a function of the number of calibration trials, showing that four trials per movement type are sufficient to achieve maximal accuracy (100%).

Figure 2 illustrates the effect of varying the number of calibration trials on classification accuracy, demonstrating that four trials per movement type achieved optimal accuracy (100%) without imposing additional burden on the user.

Following calibration, participants completed a series of guided movement sequences to assess the system’s classification accuracy. The system randomly presented 60 movement cues (20 per movement type) with timing intervals varying between 2 and 5 s to reduce rhythmic patterning. Visual feedback was provided immediately after each detected movement, showing a green checkmark for correct classification or a red “X” for misclassification. Participants could request short rest breaks if experiencing eye fatigue. Performance metrics, including accuracy, response latency, and movement consistency, were automatically logged.

**Figure 3:**
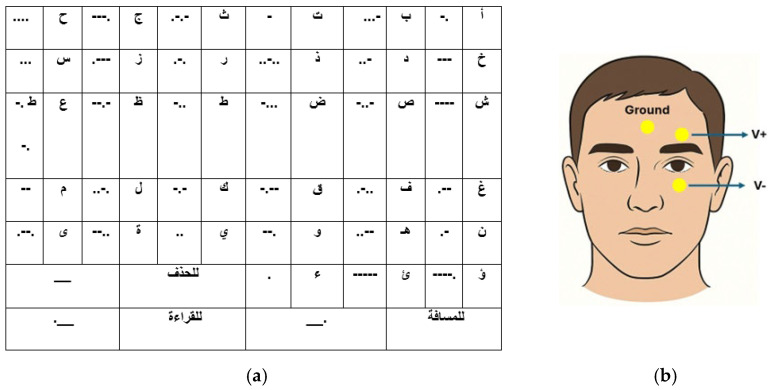
(**a**) Arabic Morse code mapping table used for text entry in the proposed EOG-based communication system. Arabic characters correspond to letters, while special labels indicate control commands, including delete (حذف), space (مسافة), and read (قراءة). (**b**) Placement of EOG electrodes for vertical eye-movement recording, showing the ground electrode on the forehead and the active electrodes positioned above (V+) and below (V−) the eye.

In the final phase, participants used the system to compose Arabic text through the Morse code interface illustrated in Figure 3a. Each letter was encoded using combinations of dots (upward eye movements) and dashes (regular blinks), with encoding lengths ranging from 1 to 5 symbols. The mapping was optimized based on Arabic letter frequency analysis, assigning shorter codes to more frequently used letters. Control commands included: space (long blink + upward movement), delete (double long blink), and text-to-speech activation (triple blink). They were given three communication tasks of increasing complexity: writing their name in Arabic (typically 4–5 characters), composing a predetermined three-word phrase (“مرحبا بالعالم”—Hello World) presented on a reference card, and creating a free-form message of their choice (with a minimum length of 10 characters).

During the communication tasks, participants could see their composed text updated in real time on the display, with the option to delete incorrect entries via a long blink. The system recorded the total time required to complete each task, the number of correction actions, and the final information transfer rate.

### 2.3. EOG Signal Acquisition and Processing

In this study, three gold-cup electrodes were used for EOG signal acquisition. Before electrode placement, the skin was cleaned using an alcohol swab, and Ten20 conductive paste (Weaver and Company, Aurora, CO, USA) was applied to ensure good electrical contact and reduce skin–electrode impedance. A ground electrode was placed on the forehead, and two recording electrodes were positioned above and below the eye to capture vertical eye-movement activity, as illustrated in Figure 3b.

**Figure 4:**
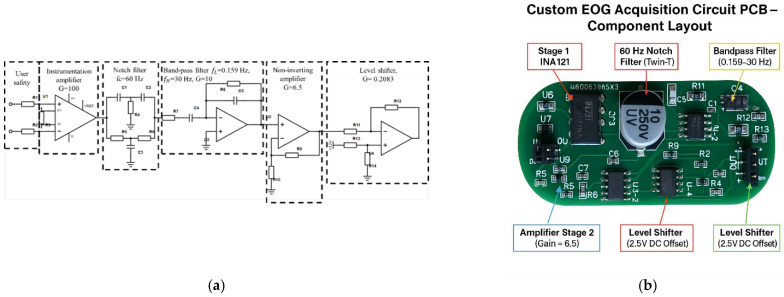
(**a**) Block diagram of the five-stage EOG acquisition circuit. (**b**) PCB layout showing the main functional stages and components.

The EOG acquisition system consisted of a five-stage analog signal-processing circuit designed to amplify and filter low-amplitude biopotential signals, as illustrated in Figure 4. The circuit addressed key challenges in EOG signal acquisition, including low signal amplitude, susceptibility to power-line interference, and baseline drift. As shown in Figure 4, the stages included an instrumentation amplifier (INA121) (Texas Instruments, Dallas, TX, USA) with a gain of 100, providing a high common-mode rejection ratio (CMRR), followed by a passive 60 Hz notch filter to suppress power-line noise. Next, a second-order Butterworth bandpass filter with cutoff frequencies of 0.159 Hz and 30 Hz was used to eliminate baseline drift and high-frequency components. A non-inverting amplifier stage with a gain of 6.5 provided further amplification. The final stage was a level-shifting circuit, which raised the baseline voltage to 2.5 V to ensure compatibility with the Arduino analog-to-digital converter (ADC) input range. The overall circuit achieved a total gain of 1428 and a signal-to-noise ratio (SNR) of 25.21 dB, which was computed in decibels using [28]:

(1) $$SNR=20{log}_{10}(\frac{V_{signal}}{V_{noise}})$$

where $V_{\mathrm{signal}}$ and $V_{\mathrm{noise}}$ are the root-mean-square (RMS) voltages of the signal and baseline-noise segments, respectively. The signal interval corresponded to the eye-movement epoch, while the noise was estimated from the resting baseline segments before and after each movement. The analog output was fed to an analog input channel of the Arduino Mega 2560 (Arduino, Ivrea, Italy), which interfaced with a Bluetooth module for wireless data transmission to the mobile application. The EOG acquisition circuit was battery-powered to minimize power-line interference.

### 2.4. Feature Extraction and Classification

Algorithm 1 illustrates the proposed eye-movement classification algorithm, which distinguishes between three eye-movement classes: blinks, long blinks, and upward eye movements. Three discriminative features were extracted from the EOG signal: peak amplitude, signal standard deviation, and event duration.

The system uses calibration-derived thresholds to distinguish between three eye-movement classes: blinks, long blinks, and upward eye movements. The classification decision logic depends on the signal peak amplitude, signal duration, and signal standard deviation. The system begins by checking whether the signal’s maximum amplitude meets or exceeds the blink amplitude threshold set during calibration. If it does not, the system evaluates whether the signal’s standard deviation is less than or equal to the blink standard deviation threshold. If so, it then checks the signal duration: if the duration is greater than or equal to the upward-movement duration threshold, the movement is classified as “Looking Up”; if the duration is less than or equal to the long-blink duration threshold, it is classified as a “Blink”; otherwise, it is classified as a “Long Blink”.

This multi-parameter approach improves upon traditional single-threshold methods by accounting for inter-individual variability in eye-movement patterns. Regular blinks are characterized by relatively high amplitude, short duration, and elevated signal variability, whereas long blinks share similar amplitude characteristics but exhibit longer durations [29]. Upward movements typically present lower amplitudes and reduced signal variability compared to blinks. The system applies user-specific thresholds determined during calibration, enabling adaptation to each user’s unique eye-movement characteristics. This personalized approach enhances overall classification accuracy across users and accommodates variability in movement strength and motor control.

A finite-state machine (FSM) converted classified movements into Morse symbols. Inter-symbol gaps longer than 200 ms indicated symbol boundaries, while gaps exceeding 2000 ms marked character completion. Decoded Arabic characters were displayed in real time with visual feedback and optional text-to-speech output, enabling immediate error detection and correction.
**Algorithm 1.** Adaptive classification of EOG-based eye movements using threshold-based logic.**1. Procedure** **2. Input:** signal_max, signal_std, signal_samples, signal_duration
**3.**    blink_threshold, blink_std, up_std, up_sample, long_duration
**4. if** signal_max > blink_threshold **then**
**5.    if** signal_std > up_std and signal_duration < long_duration **then**
**6.**      Movement_Type ← Blink
**7.    else if** signal_std > up_std **then**
**8.**      Movement_Type ← Long Blink
**9.    else if** signal_samples > up_sample **then**
**10.**      Movement_Type ← Upward Movement 
**11.    else**
**12.**      Movement_Type ← Long Blink 
**13.    end if**
**14. else**
**15.    if** signal_std < blink_std or signal_samples > up_sample **then**
**16.**      Movement_Type ← Upward Movement
**17.    else if** signal_duration < long_duration **then**
**18.**      Movement_Type ← Blink 
**19.    else**
**20.**      Movement_Type ← Long Blink 
**21.    end if**
**22. end if**
**23.**
**24. return** Movement_Type
**25. end procedure**


## 3. Results

### 3.1. Performance Evaluation of the Custom EOG Acquisition Circuit

The custom-designed EOG acquisition circuit demonstrated clear performance advantages over commercial alternatives, such as the OpenBCI Cyton board (OpenBCI, Brooklyn, NY, USA). Specifically, it achieved a signal-to-noise ratio (SNR) of 25.21 dB, representing an approximately 8% improvement over the 23.34 dB obtained using the OpenBCI Cyton board. Moreover, the total hardware cost for the custom-designed circuit was USD 152, representing an 87.9% cost reduction compared to the OpenBCI’s price of USD 1249. Additionally, the custom circuit maintained low power consumption, operating at 45 mW. This substantially outperforms typical commercial systems in energy efficiency, which is critical for both long-term usability and patient comfort.

**Figure 5:**
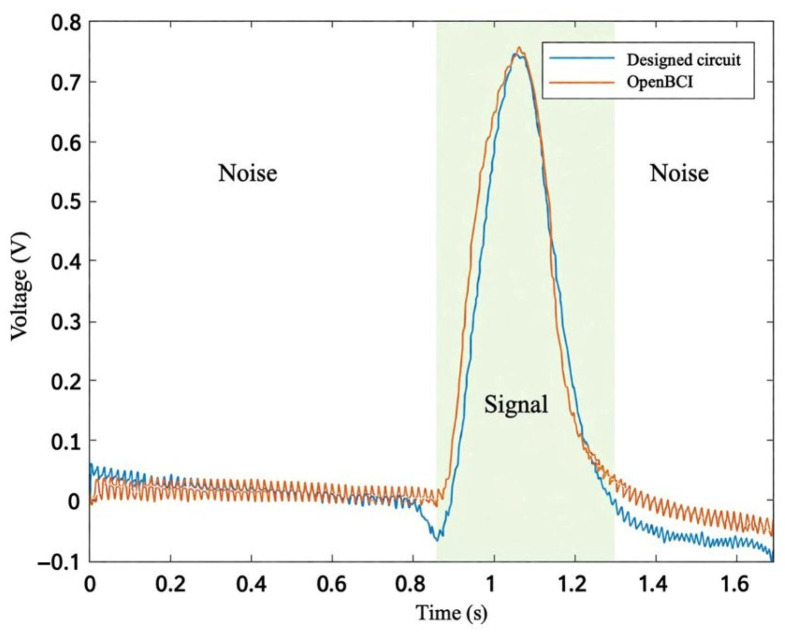
Comparison of EOG signals acquired from the same subject using the custom-designed circuit and the OpenBCI Cyton board.

Figure 5 illustrates a direct visual comparison of EOG signals recorded simultaneously using the custom-designed circuit and the OpenBCI Cyton board. As shown, the waveform recorded from the custom circuit demonstrates lower baseline noise and higher peak amplitudes, reflecting the superior signal-to-noise ratio (SNR) achieved by the proposed design.

### 3.2. Classification Performance

**Figure 6:**
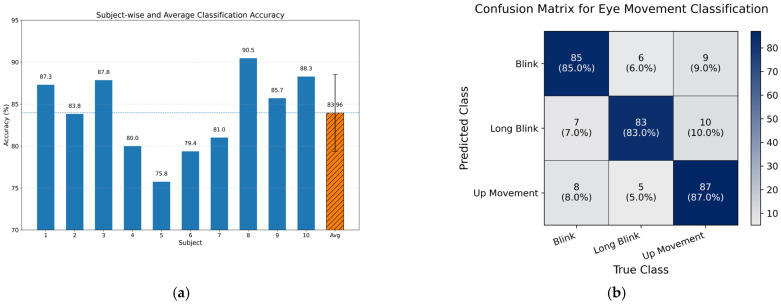
(**a**) Classification accuracy achieved by each of the ten subjects (blue bars), along with the overall mean accuracy (hatched orange bar, 83.96% ± 4.59%). (**b**) Confusion matrix summarizing the classification performance of the proposed adaptive algorithm across all participants (*n* = 100 trials per movement type), showing movement-specific accuracies of 85% for blinks, 83% for long blinks, and 87% for upward movements.

Testing with 10 subjects yielded an average accuracy of 83.96% ± 4.59%. Individual accuracies ranged from a minimum of 75.8% to a maximum of 90.5%, as shown in Figure 6a, indicating inter-subject variability in performance. Notably, classification performance improved as participants became familiar with the system, with measurable gains observed even within the first testing session. Figure 6b presents the confusion matrix of the three eye-movement classes, summarizing the classification performance of the proposed adaptive algorithm across all participants. Movement-specific accuracies were 85% for blinks, 83% for long blinks, and 87% for upward movements.

### 3.3. Communication Efficiency and Usability

**Figure 7:**
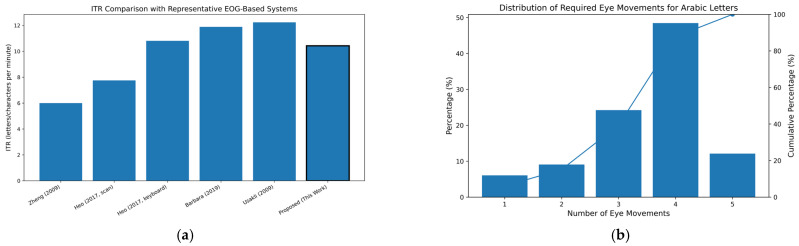
(**a**) Comparison of information transfer rate (ITR) with representative EOG-based communication systems from the literature, including Zheng et al. [31], Heo et al. [30], Barbara et al. [11], and Usakli and Gurkan [12]. (**b**) Distribution of the number of eye movements required for Arabic letter entry, along with cumulative percentage. Approximately 90.9% of letters require four or fewer movements, indicating efficient interaction and reduced user effort.

The proposed system achieved an information transfer rate (ITR) of 10.43 letters per minute, as shown in Figure 7a. This performance is comparable to modern EOG-based virtual keyboard systems, such as that presented by Heo et al. [30], which reported 10.81 letters per minute, and that by Barbara et al. [11], which achieved 11.89 characters per minute. The proposed system also outperforms simpler portable implementations, such as those of Zheng et al. [31], which report approximately 6 letters per minute. Although the achieved ITR is slightly lower than that reported by Usakli and Gurkan [12] (12.24 letters per minute), the proposed system offers improved usability and reduced movement complexity, making it more practical for real-time communication.

The average response time between eye movement detection and letter display was 2.01 s, reflecting the system’s responsiveness during real-time interaction. The movement requirements were analyzed across the full Arabic alphabet. The results show that 6.06% of letters require one movement, 9.09% require two movements, 24.24% require three movements, 48.48% require four movements, and 12.12% require five movements, as illustrated in Figure 7b. Based on this distribution, approximately 90.9% of letters can be generated using four or fewer movements, indicating reduced user effort and improved communication efficiency. The results indicate that the proposed system achieves competitive ITR while requiring fewer movements for the majority of characters. This reflects an improved balance between communication speed and interaction simplicity compared to existing systems.

## 4. Discussion

This study demonstrates several improvements in EOG-based communication systems tailored for patients with LIS. It addresses critical limitations observed in existing solutions, including limited usability, lack of language-specific support, and reliance on high-cost hardware.

The superior signal-to-noise ratio (SNR) achieved by the custom-designed circuit indicates enhanced signal quality with reduced noise interference, which is essential for accurate eye-movement detection. The improved SNR is mainly attributed to the front-end instrumentation amplifier combined with carefully tuned notch and bandpass filters, which effectively suppress power-line interference and out-of-band noise while preserving the EOG signal. The overall performance is further influenced by design choices such as distributing gain across multiple stages and selecting appropriate filter bandwidths, which help prevent saturation and reduce noise amplification.

Moreover, the substantial cost reduction improves accessibility in both clinical and home-based environments, addressing a major barrier to the adoption of assistive technologies. Compared to general-purpose systems such as OpenBCI, the proposed design is specifically optimized for EOG acquisition, enabling comparable signal quality with lower cost and reduced system complexity.

The proposed multi-parameter classification strategy effectively addresses limitations found in previous systems. For instance, threshold-based systems, such as that of Banik et al. [11], achieve high accuracy under controlled conditions but exhibit limited robustness to varied movement patterns. In contrast, the proposed approach achieved an average accuracy of 83.96% ± 4.59% while providing greater adaptability to individual variability.

The observed learning effect among first-time users highlights the importance of the calibration and familiarization process. Common errors, such as unintended eyebrow movements or incomplete returns to neutral gaze, decreased with practice, indicating the potential for improved performance with continued use.

The successful implementation of Arabic Morse code demonstrates an effective approach for language-specific communication systems. A 30.38% improvement in writing speed compared with traditional keyboard-based methods indicates the practical utility of the system, particularly when combined with the auto-complete feature.

The system’s performance metrics and design features suggest its potential as a candidate for clinical evaluation. Its portability, affordability, and user-adaptive design address key barriers to technology adoption in healthcare settings. The simple electrode placement and straightforward calibration procedure can be performed by healthcare providers with minimal specialized training, facilitating integration into existing care workflows.

For patients with LIS, the system may offer potential improvements in quality of life by enabling autonomous communication, which has been associated with reduced depression and improved psychological well-being in prior studies. The inclusion of Arabic language support addresses an unmet regional need, potentially reducing linguistic barriers in patient–provider communication.

The system’s adaptive calibration framework allows it to accommodate varying levels of eye-movement control, making it suitable for patients at different stages of LIS or with differing degrees of motor impairment. This adaptability may extend its applicability to patients with other communication-limiting conditions that preserve eye movement, such as advanced ALS, cerebral palsy, or post-stroke impairments.

In transitioning from healthy subjects to real-world clinical applications, it is essential to consider the additional signal variability present in patients with Locked-in Syndrome (LIS). While LIS is primarily characterized by paralysis of voluntary muscles, patients may still exhibit involuntary muscle activity, such as facial tremors, spasticity, or sustained tension in the periocular and masticatory regions [4]. These factors can introduce baseline drift, increased signal variability, and high-frequency transient artifacts, potentially affecting the reliability of EOG-based classification.

The proposed system incorporates several mechanisms to mitigate these challenges. First, the custom-designed acquisition circuit achieved a high signal-to-noise ratio (SNR) of 25.21 dB, which enhances the system’s ability to distinguish voluntary eye movements from background physiological noise. The analog front-end employs notch and bandpass filtering (0.159–30 Hz) to suppress power-line interference and attenuate high-frequency components, including electromyographic (EMG) artifacts associated with muscle tension. Second, the hybrid classification approach, combining amplitude, temporal, and statistical features, enhances robustness by requiring multiple feature conditions to be satisfied simultaneously, thereby reducing susceptibility to noise.

To address unintended signals or misclassified inputs, the system incorporates real-time visual feedback along with user-initiated correction commands (e.g., deletion via long blink), enabling active error correction during operation. In addition, future implementations may benefit from incorporating confidence-based classification, temporal consistency constraints, and rejection of ambiguous or low-confidence events, which are particularly important in minimizing false activations in clinical populations. These considerations support a more robust and patient-centered system design. Although these strategies improve robustness, clinical validation remains essential to fully characterize system performance under patient-specific conditions.

This study was conducted on healthy subjects and therefore represents a preliminary evaluation of the proposed system; further validation on LIS patients is required to confirm clinical applicability. Future work should include clinical validation on LIS patients and longer-duration testing to assess system reliability during prolonged use, as fatigue-related effects may influence eye-movement control over time. Additionally, integration with hospital information systems could further enhance clinical utility by enabling direct patient input into electronic health records and remote communication with healthcare providers.

## 5. Conclusions

This paper presented an optimized EOG-based communication system specifically designed for Arabic-speaking patients with locked-in syndrome. The system addresses three core challenges in assistive communication technologies: cost-effectiveness, classification accuracy, and Arabic language support. The hardware implementation demonstrated that high-quality biomedical signal acquisition can be achieved at significantly lower costs than commercial alternatives. The custom-designed circuit achieved an 8% improvement in the SNR while reducing costs by 62%. The adaptive classification algorithm proved effective across different users, with an average accuracy of 83.96% ± 4.59% that improved with user familiarity. By combining multiple signal parameters (amplitude, duration, and standard deviation), the system accommodates individual variations in eye movement patterns, a critical factor for practical deployment. The integration of Arabic language support through Morse code, coupled with auto-completion and text-to-speech capabilities, resulted in communication speeds of 10.43 letters/minute, representing a 30.38% improvement over traditional methods. This demonstrates the system’s potential to improve communication; however, validation on LIS patients is required to confirm clinical applicability. Future work will focus on implementing AI-enhanced word prediction, expanding platform support beyond Android, and conducting extended testing with LIS patients in clinical settings. These enhancements will further improve the system’s performance and accessibility for those who need it most.

## Figures and Tables

**Table 1:** Comparison of representative EOG-based communication systems and the proposed system. NR = not reported; CER = character error rate; lpm = letters per minute. SNR is rarely reported; the proposed system provides an explicit value (25.21 dB).

Ref.	Target Users	Application	Language/Script	Accuracy (%)	ITR (lpm)	SNR (dB)
[12]	Healthy subjects	Virtual keyboard for text entry and basic needs selection	Not specified (alphanumeric)	95	~12 *	NR
[13]	Healthy subjects	EOG-based writing system (text generation with GUI)	Not specified (alphanumeric system)	~98 *	~20 *	~20 †
[14]	Healthy subjects	Message selection through 10 predefined buttons using single-channel horizontal EOG; output shown on LCD	Not specified (alphanumeric)	92.0–99.6	NR	NR
[15]	Healthy subjects and a patient with movement disability	5-option selection-based communication (gaze + blink)	Not specified (fixed-word menu)	100 (healthy), ~60 (patient)	NR	NR
[16]	Healthy subjects	Continuous eye-writing (trajectory-based character input)	Japanese (Katakana)	~95 (CER = 5.0%)	27.9	NR
Proposed system	Healthy subjects	Arabic EOG-based communication system (blink + vertical eye movement; Morse-like encoding)	Arabic	83.96 ± 4.59	10.43	25.21

* Values estimated or derived from reported figures or experimental descriptions, as explicit numerical values were not provided in the original studies. † SNR value refers to signal processing (denoising stage) and not acquisition-level SNR.

## Data Availability

The data presented in this study are not publicly available due to ethical and privacy restrictions related to human subject research. De-identified data may be made available from the corresponding author upon reasonable request and subject to approval by the Institutional Review Board.

## Abbreviations

The following abbreviations are used in this manuscript:

EOG	Electrooculogram
LIS	Locked-in Syndrome
SNR	Signal-to-Noise Ratio
ITR	Information Transfer Rate
ADC	Analog-to-Digital Converter
CMRR	Common-Mode Rejection Ratio
FSM	Finite-State Machine
ALS	Amyotrophic Lateral Sclerosis
POG	Photo-Oculography
VOG	Video-Oculography
